# Algoritmically improved microwave radar monitors breathing more acurrate than sensorized belt

**DOI:** 10.1038/s41598-022-18808-2

**Published:** 2022-08-24

**Authors:** Andrzej Czyżewski, Bozena Kostek, Adam Kurowski, Krzysztof Narkiewicz, Beata Graff, Piotr Odya, Tomasz Śmiałkowski, Andrzej Sroczyński

**Affiliations:** 1 Department of Multimedia Systems, Faculty of Electronics, Telecommunications and Informatics, Gdańsk University of Technology, 80‑233 Gdańsk, Poland; 2 Audio Acoustics Department, Faculty of Electronics, Telecommunications and Informatics, Gdańsk University of Technology, 80‑233 Gdańsk, Poland; 3 Department of Hypertension and Diabetology, Medical University of Gdańsk, 80‑210 Gdańsk, Poland; 4 Siled Co. Ltd, 83-011 Gdańsk, Poland

**Keywords:** Engineering, Biomedical engineering

## Abstract

This paper describes a novel way to measure, process, analyze, and compare respiratory signals acquired by two types of devices: a wearable sensorized belt and a microwave radar-based sensor. Both devices provide breathing rate readouts. First, the background research is presented. Then, the underlying principles and working parameters of the microwave radar-based sensor, a contactless device for monitoring breathing, are described. The breathing rate measurement protocol is then presented, and the proposed algorithm for octave error elimination is introduced. Details are provided about the data processing phase; specifically, the management of signals acquired from two devices with different working principles and how they are resampled with a common processing sample rate. This is followed by an analysis of respiratory signals experimentally acquired by the belt and microwave radar-based sensors. The analysis outcomes were checked using Levene’s test, the Kruskal–Wallis test, and Dunn’s post hoc test. The findings show that the proposed assessment method is statistically stable. The source of variability lies in the person-triggered breathing patterns rather than the working principles of the devices used. Finally, conclusions are derived, and future work is outlined.

## Introduction

In-hospital and out-of-hospital diagnostic and therapeutic processes could be improved by utilizing existing innovative microelectronic and information technology (IT)-based systems to record and analyze vital signs. Currently, a range of commercially available devices and applications are widely used on a daily basis to monitor the respiratory rate, and they are often used during sporting activities and to monitor patients and elderly people at home^1^. Unfortunately, not all such devices and applications provide a reliable assessment of the breathing rate. However, there has been significant progress made by the medical and scientific communities in the development of methods and techniques for respiratory monitoring^1–3^. Two recent papers have comprehensively reviewed several such devices based on their working principles^2,3^, which include acoustic (respiratory sounds), resistive (respiratory activity causing changes in air humidity), optic (fiber-optic-based air flowmeters), inductive and pressure (chest wall movements), humidity/temperature, acceleration (flow measurement and chest wall movements), electromyography (biopotentials), impedance (chest wall strains), infrared (gaseous absorption), and microwave^4–8^. Most of the technologies are non-intrusive, contact-based solutions. However, since the emergence of the coronavirus disease (COVID-19) pandemic, it has become essential to develop contactless devices. The use of devices that do not contact the patient’s body reduces the need for disinfection procedures and is safer for both patients and medical staff.

The aim of this study was to compare the respiratory activity measurement acquired by a standard wearable device (a belt) and a contactless device developed by the authors. We aimed to develop a low-cost radar-based solution that can be used in both clinical settings and patients’ homes to diagnose and monitor patients with respiratory problems, most notably patients with contagious diseases such as COVID-19. Hence, parameters critical for non-invasive, contactless, automatic respiratory monitoring were assessed in this study. The proposed solution is the result of close collaboration between the Department of Multimedia Systems of the Faculty of Electronics, Telecommunications and Informatics, Gdańsk University of Technology, and the Department of Hypertension and Diabetology of the Medical University of Gdańsk in Poland.

First, a brief review of available sensor technology and corresponding signal processing suitable for monitoring respiration is presented. Then, our novel approach to monitoring breathing rate using a contactless microwave radar-based sensor is presented, which employs an algorithm for octave error elimination. The experimental protocol is followed by a description of the method used by the radar-based sensor to acquire respiratory signals and how they are compared with the signals obtained from the standard wearable belt. Finally, the results are discussed and conclusions drawn, and potential applications of the engineered solution are outlined.

## Respiratory rate measurement devices

### State-of-the-art

In the literature, several devices are described as the gold standard in terms of respiratory rate measurement and airflow limitation^9^. These devices are used for spirometry (the accurate and repeatable measurement of lung function) and capnography (which enables evaluation of a patient’s metabolism, ventilation, and perfusion). However, a plethora of breathing rate measurement methods, devices, and technologies exist^1–3,10,11^, most of which are contact-based. They include the following: infrared cameras and infrared thermography^12–15^; video cameras, especially with motion magnification algorithms^16–18^; fiber-optic *s*ensors^19^; light intensity sensors^20^; thermal imaging^21^; thermistors^22^; inductive sensors^23^; impedance (transthoracic) sensors^24^; ultrasonic transmitters and receivers^25^; movement sensors (accelerometers, gyroscopes, magnetometer)^26–28^; and microwave based on the Doppler effect (a comprehensive review is given in^29^).

These devices have been applied in several clinical settings. For example, sleep apnea can be diagnosed using respiratory effort-based signals, and a comparative study in this domain is reported in^30,31^. A non-invasive Wi-Fi-based breathing estimator was proposed by Abdelnasser et al.^32^. A similar method, employing Wi-Fi frame capture, was developed by Kanda et al.^33^, and Ghafar-Zadeh et al. described a new approach that may be applied to free-breathing spirometry based on microsensors that can be integrated into mobile phones for lung care purposes^34^. However, their work concentrated on assembling the device rather than on clinical trials. It should also be noted that a thorough and reliable respiratory and lung function assessment is essential before withdrawing mechanical ventilation^35^.

Therefore, it may be concluded that there is a clear need in many medical in-hospital and out-of-hospital situations for reliable contactless patient evaluation. Here, we propose and describe an algorithmic-based approach to respiratory pattern assessment based on a contactless microwave radar-based sensor.

## The Respiration and Circulation Monitor

**Figure 1:**
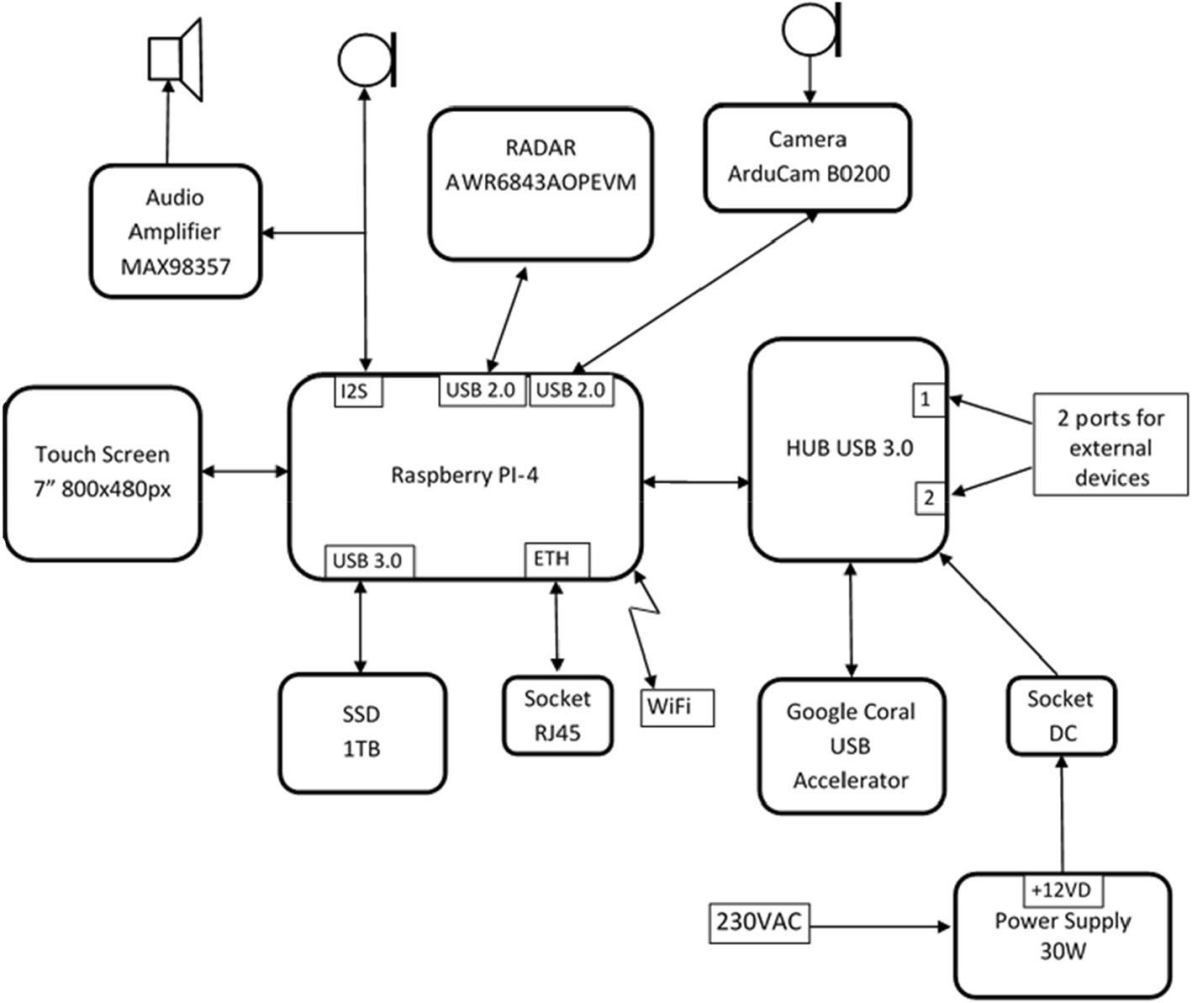
Block diagram of the components of the Respiration and Circulation Monitor.

**Figure 2:**
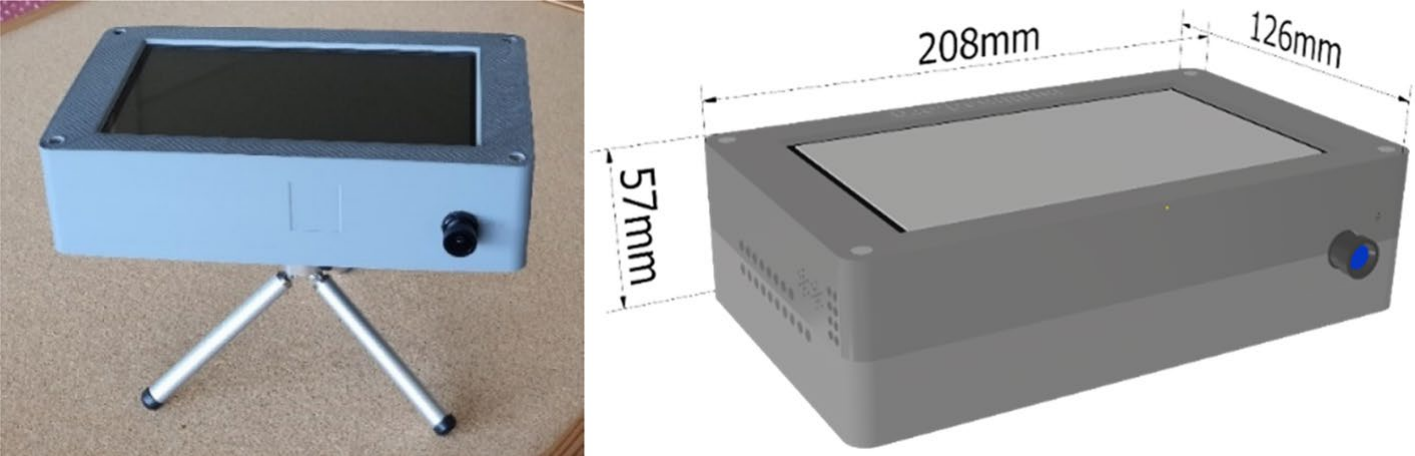
External appearance and dimensions of the Respiration and Circulation Monitor.

Our Respiration and Circulation Monitor is a stand-alone radar-based sensor device employing a Raspberry PI-4 microcomputer card with sensors (i.e., radar, camera, and microphone) and a 7″ touch screen connected to the card. In addition, the device is equipped with a standard Ethernet interface, a Wi-Fi module to connect to the cloud application, and two USB 3.0 ports to couple the external systems. Figure 1. is a block diagram that shows the device’s components. The engineered device and its dimensions are shown in Fig. 2.

The millimeter-wave radar is a particular type of device that emits short-wavelength electromagnetic waves and then records the waves reflected by the object under study. This solution has been successfully employed in the automotive industry to detect free space in the cabin and obstacles near the doors and trunk, as an intelligent automatic parking system or intruder alarm, to warn against leaving a child in a parked car, and for other uses. Given that this state-of-the-art technology is highly accurate in detecting movements of fractions of a millimeter, we used this technology to construct a device to detect chest movements in a contactless manner. Also, heart rhythm can be acquired by this device. It should be noted that it is necessary to obtain a declaration from the sensor’s manufacturer to ensure compliance of the device’s components with the safety standards of the European Union^36^.

The radar module was realized using a single-chip IWR6843AOP sensor from Texas Instruments, operating at 60–64 GHz. The module contains built-in Antennas-On-Package (AOP) transmitting and receiving antennae. Additionally, the radar is equipped with a Digital Signal Processing (DSP) subsystem based on a TI C674x chip, which is used in algorithms that process data from the radar^36,37^. The firmware for the Respiration and Circulation Monitor is based on the VitalSigns project and provided by the manufacturer in the Industrial Toolbox package included in Software development kit (SDK) for the IWR6843AOPEVM chip^36^. The software in the radar firmware pre-processes the antenna data in the frequency domain using Fourier transform enabled by an integrated digital signal processing subsystem with a radar hardware accelerator^38^. However, other approaches to managing antenna data, such as ellipse reconstruction, have been reported in the literature^39–41^. The project was modified regarding session repetition while maintaining the radio chip settings. Also, an algorithm developed at the Gdańsk University of Technology has been implemented in the signal processor code to remove octave errors appearing in the device output signal. The octave errors here represent readings that indicate precisely twice the actual respiratory rate, hence the need to eliminate them.

The Coral PoseNet project^42,43^ is used to detect the correct body layout for the measurement. This application is based on the MobileNet V1 model^44^. A convolutional neural network processes the camera stream. The Google Coral USB Accelerator—Edge TPU ML—ARM Cortex M0 is employed as the machine learning accelerator. In particular, the TensorFlow Lite platform dedicated to embedded devices supports deep learning inference mechanisms. The application for the operation of the Respiration and Circulation Monitor was created in Python version 3.7.3. The application is used to operate the device, that is, to read the data from the radar, convert it into numerical form, save it into a measurement file, and display the respiratory waveform (and pulse) on the device screen.

Finally, an essential feature of the device should be the automated assessment of the breathing pattern. There are a few examples where machine learning-based analysis has been used to detect breathing abnormalities or cessation^45–48^. It is appropriate to recall Szczuko et al.’s work here, as it is considered a cognitive approach to respiratory data curation and analysis^48^. Automatic classification of the breathing pattern is a very promising achievement that should be pursued in future work.

## Experimental protocol

### Materials

The study group consisted of 31 healthy volunteers (11 men) with no signs of dyspnea. Their mean age was 42 years (range: 20–66 years), and their mean weight was 81 kg (range: 55–115 kg). The study was conducted at the Medical University of Gdańsk. The study complied with the Declaration of Helsinki, and the protocol was approved by the Ethics Committee of the Medical University of Gdańsk (NKEBN/422/2011). All participants were informed about all the details of the trial and the study’s merits. They also signed written consent forms. While the group consisted of healthy volunteers, it was highly diverse, as the trial aimed to determine whether any anomalies in the measurement results might occur. All data gathered were anonymized.

### Trial methodology and protocol

Each individual’s respiratory pattern was recorded using the respiratory belt (TN1132/ST), which was connected to the PowerLab system with the proprietary Lab Chart 8 software (ADInstruments, Australia)^49,50^. The respiratory belt transducer is designed to measure chest (or abdomen) circumference changes resulting from breathing. The sampling rate was 1,000 Hz. The respiratory sensorized belt produced a linear voltage within the range of 0 to 100 mV proportional to changes in length. As a result, it was possible to derive the respiratory pattern. At the same time, an electrocardiogram was recorded and a blood oxygen saturation (SpO_2_) measurement was performed. The abovementioned hardware and software are standard equipment in research laboratories designed for studying cardiovascular and respiratory regulation.

**Figure 3:**
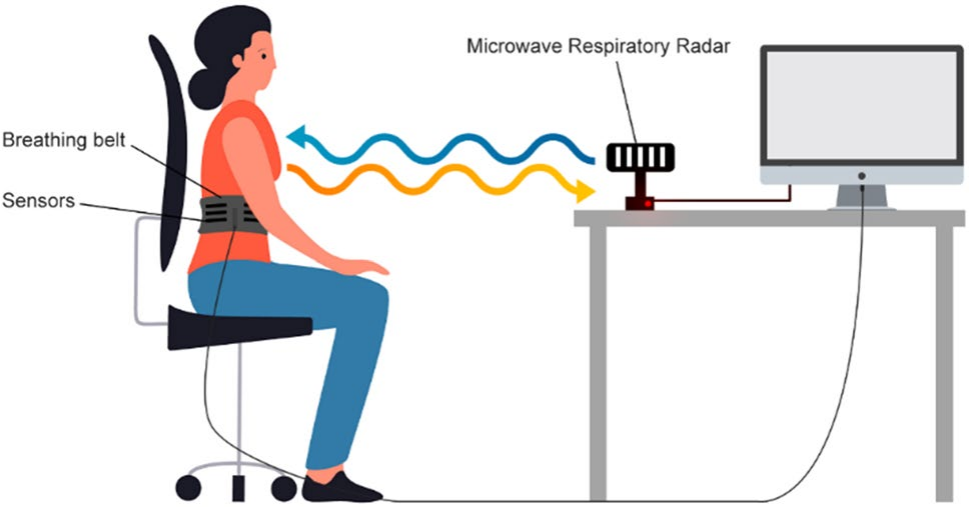
The experimental set-up.

For the experimental part of the study, each participant wore a respiratory belt, and the radar sensor was placed approximately 1 m in front of them at chest height. Controlled regular breathing was achieved with the help of custom-made software that produces different sounds for inspiration and expiration at the intended frequency. Figure 3 shows the experimental set-up. A clinician was always present during the measurement to ensure that the trial participant was correctly positioned relative to the radar sensor. Hence, any problems associated with the movement of the participants may not have been captured for later consideration. Furthermore, the software that processes the raw radar data is engineered to reject the type of data segments that large-amplitude movements might produce.

The study protocol consisted of the following three parts: 10 min of recording during spontaneous breathing, 5 min of recording during controlled respiration at a frequency of 12 breaths/min, and 5 min of recording during controlled respiration at a frequency of 15 breaths/min.

## Method

### Data processing and analysis

**Figure 4:**
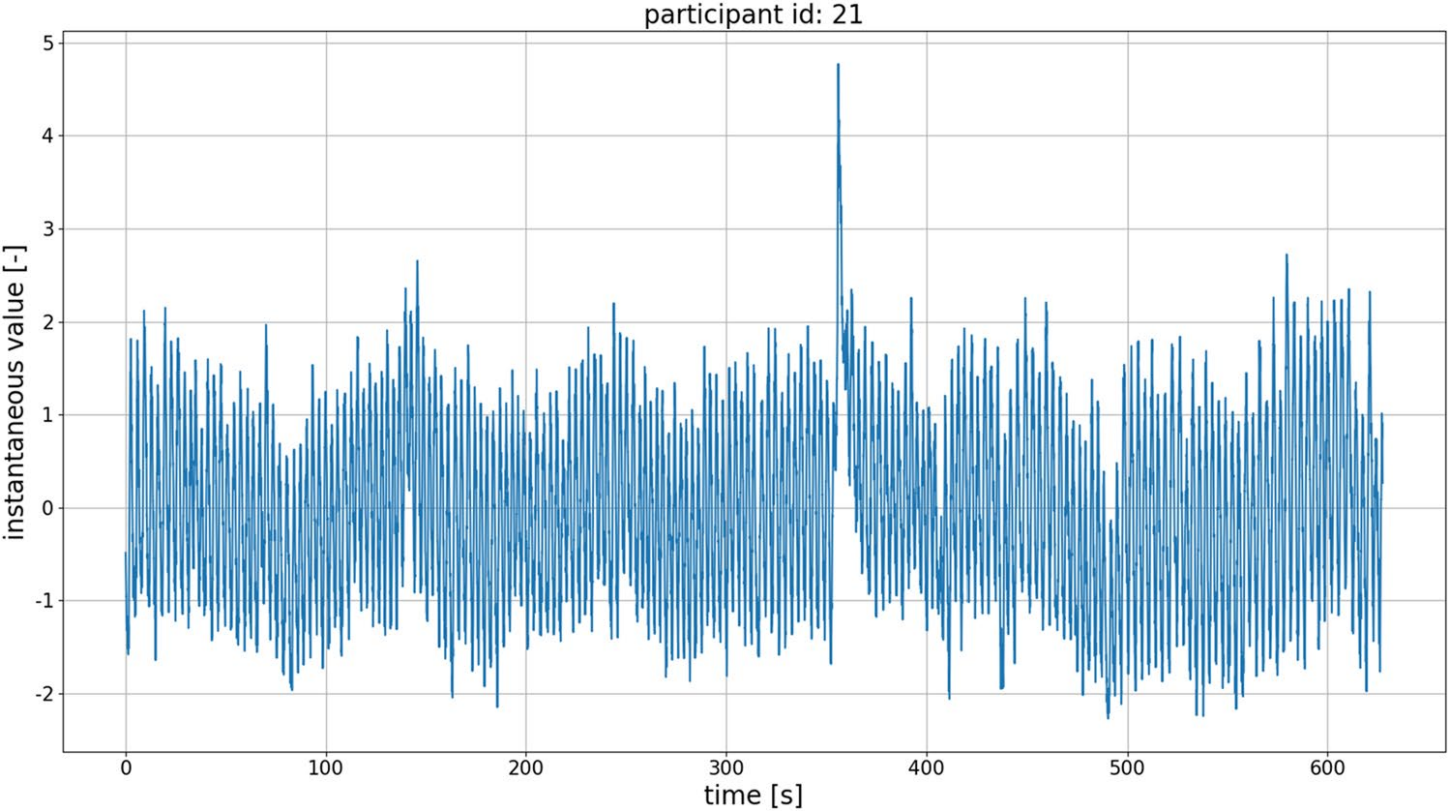
Example of the respiratory signal obtained from Participant 21 using the belt device.

The input data for the algorithm was provided in two formats: the .csv format for data obtained using the microwave-based radar sensor and the .adicht format for data obtained using the mechanical belt sensor. Data were processed using scripts written in the Python programming language. For the reading and interpretation of the .adicht files, the adi package available on GitHub was used^51^. The original sampling rate of the belt-associated signal was 1000 Sa/s, and the sampling rate of the microwave radar-associated signals was 30 Sa/s. The signals were resampled using the common sample rate of 100 Sa/s. In the case of downsampling, the antialiasing low-pass filter based on the third-order Butterworth filter was applied. Filtering was performed using the *filtfilt* function provided in the SciPy library (version 1.5.4), which performs the filtering twice. The first pass is traditionally applied to the signal; then, the signal is reversed, and filtering is performed again. This mitigates any frequency-dependent time delays of signals to which the filtration process is applied. An example of the respiratory signal gathered by the belt device from Participant 21 is shown in Fig. 4.

Next, the data were split into frames of 4,096 samples, which corresponded to 40.96 s of data acquisition. Hence, if a subject performs large movements in the first 40 s of recording, there is a risk that the algorithm will make an error. However, this is a relatively long time, and the positions recorded by the radar are averaged during the calculation. In consequence, the selection of an incorrect trajectory is unlikely if the measurement is performed by a trained person who is aware of the fact that the subject should try to remain in relative stillness. Frame overlapping was applied to increase the time resolution of the analysis. An overlap coefficient of 0.85 was used, giving an effective temporal resolution of the analysis of 6.144 s. The resultant signal sample frames were used as the input for the algorithm used for respiratory rate estimation.

Further on, for statistical data analyses, procedures from SciPy (version 1.5.4) and statsmodels (version 0.12.2) Python libraries were employed.

### Breathing rate estimation algorithm

**Figure 5:**
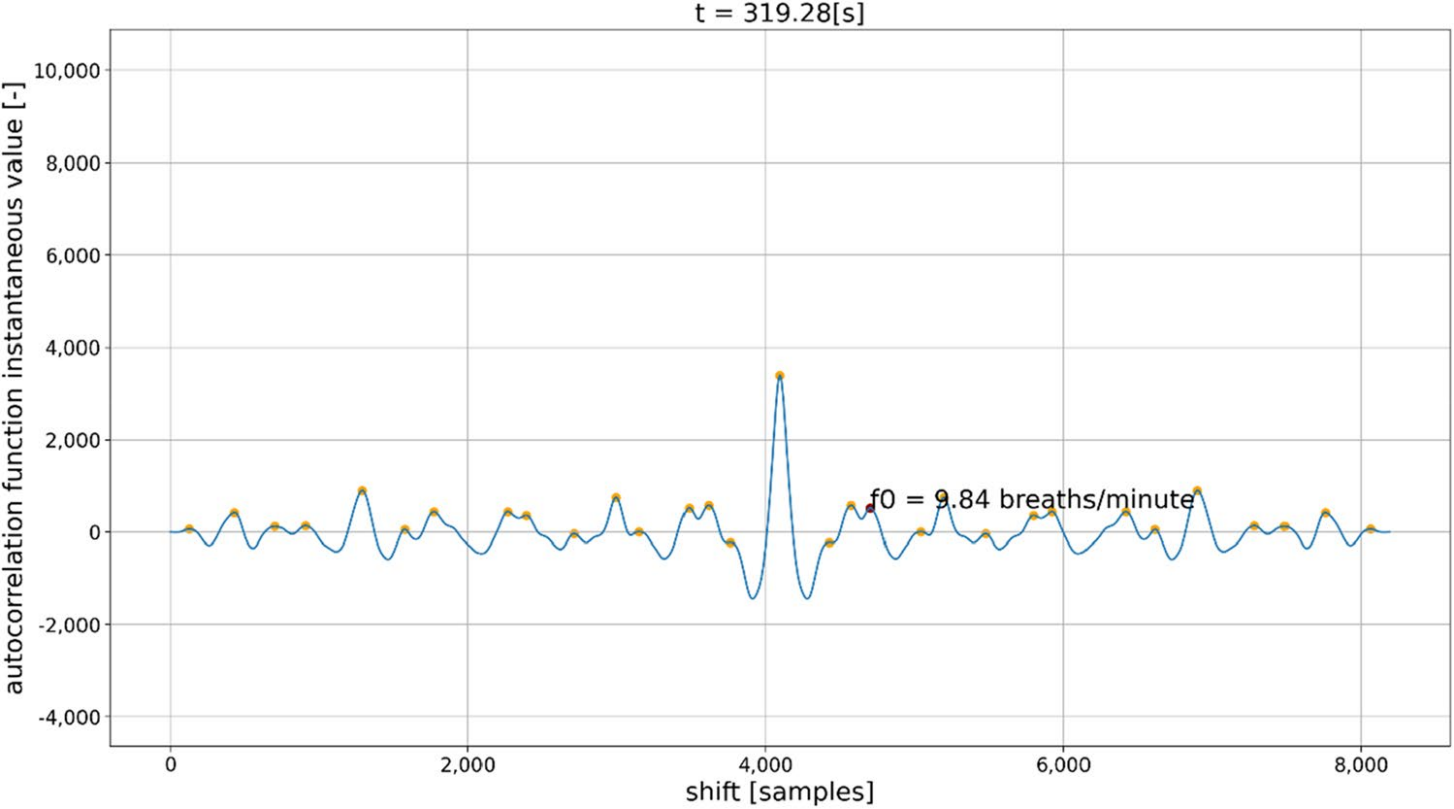
Example of the autocorrelation function for which the maximum related to the fundamental signal frequency had to be obtained by tracking a consecutive series of data frames. If there was no temporal context, then a neighboring peak to the left of the central peak would have been chosen as the second-largest maximum in the depicted autocorrelation function.

To estimate the respiratory rate from the signals obtained from the belt and the microwave radar-based sensors, detection of the signal’s fundamental frequency was necessary. To achieve this goal, the frames of the raw input signals were used to calculate their autocorrelation function. Next, for each autocorrelation function, all the maxima were localized by calculating the derivative of the autocorrelation function, and the indices were stored for further processing. In the simplest scenario, it would be sufficient to find the second-highest maximum of the autocorrelation and calculate the fundamental component period and thus the resulting fundamental frequency. However, for the signals obtained from the belt sensor and especially from the microwave radar sensor, it was found that the required fundamental frequency information was generally not available in the second-highest maximum of each frame. Hence, there were many spurious detections, which resulted in octave errors made by the naïve algorithm. An example of the type of autocorrelation function that confused the algorithm based on the simplistic principle of obtaining the fundamental frequency estimate is shown in Fig. 5.

Hence, the algorithm used to calculate the breathing rate needed to consider that, in some cases, the fundamental frequency information obtained from the second maximum of the autocorrelation function may have been invalid.

**Figure 6:**
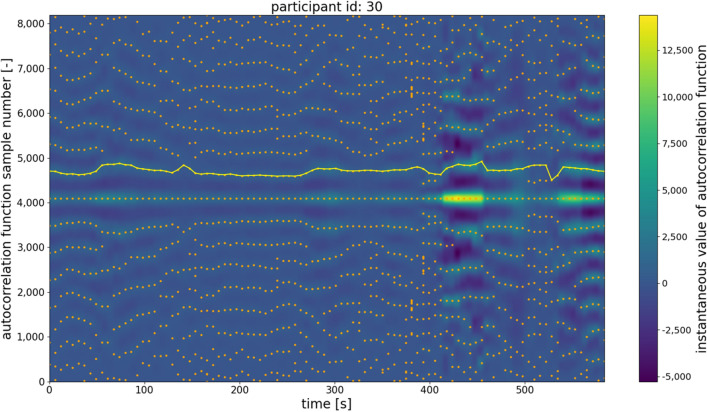
Example of the autocorrelation second-largest maximum trajectory computed by the proposed algorithm. The peaks derived from the belt-based device are better separated; however, there are some frames in which an octave error could occur if the analysis does not include the whole trajectory of the second maximum.

**Figure 7:**
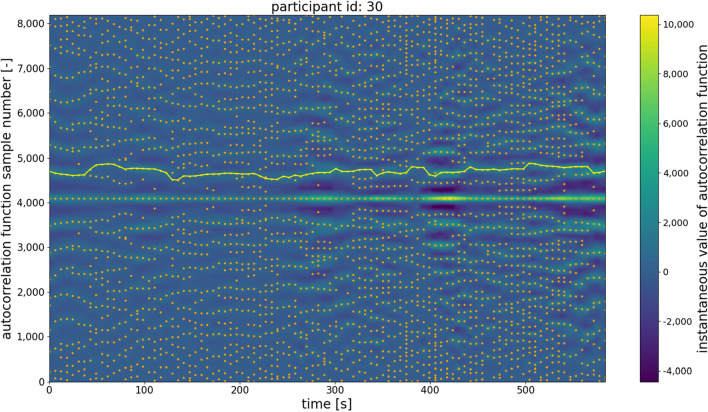
Example of the autocorrelation second-largest maximum trajectory obtained for the signal from the microwave-based radar sensor. Several problematic frames contain only a weak maximum related to the signal fundamental frequency. Moreover, many closely positioned maxima are observed, which makes tracking the fundamental frequency more challenging than in the case of the signal derived from the belt-based device.

Even though such a situation was rare, the frequency was found to be high enough to create considerable octave errors if not addressed. Therefore, it was determined that the second-largest maximum index across the whole temporal trajectory must be estimated. This process also needed to include the operations of finding unlikely fundamental frequency estimates and attempting to correct them to more probable values. Examples of such trajectories are shown in Figs. 6 and 7. As shown in Figs. 6 and 7, each vertical row of image pixels corresponds to one autocorrelation function (extracted from a single signal frame). Hence, the line near sample #4000 represents the central maxima of the listed autocorrelation functions. Since the frames were all the same length, the maxima are arranged in a straight line, and this is also the symmetry line of the image (the top half mirrors the bottom half). Highlighted in yellow is the trajectory determined by the second autocorrelation maximum, which contains useful information about the fundamental (breathing) frequency.

The general structure of the respiratory rate estimation algorithm is based on Eq. 1 and is shown in Pseudocode 1. The pseudocode syntax is based on Python language syntax. The algorithm’s pre-processing step consists of splitting the input signal into frames of a given length (specified by the frame_length variable). For each frame, an autocorrelation function is calculated. This autocorrelation function is used to calculate indices of autocorrelation function maxima. A set of such maxima vectors is called a maxima lattice (in Pseudocode 1, it is associated with a maxima_lattice variable). This maxima_lattice variable is the input for all latter procedures used to estimate the fundamental period for each frame.

The samples of the analyzed signal frame, denoted by the frame length $N$ and the number of frames *L*, are as follows:

1 $${x}_{l}=\left[{x}_{l}\left(1\right),{x}_{l}\left(2\right),..,{x}_{l}\left(N\right)\right],$$

where the index $l=1..L$. The autocorrelation of the frame $l$ is calculated using the following formula:

2 $$\phi (\tau )=\frac{1}{N}\sum_{n=0}^{N-1}x\left(k\right)x\left(n+\tau \right),$$

where $\tau$ is the lag number.
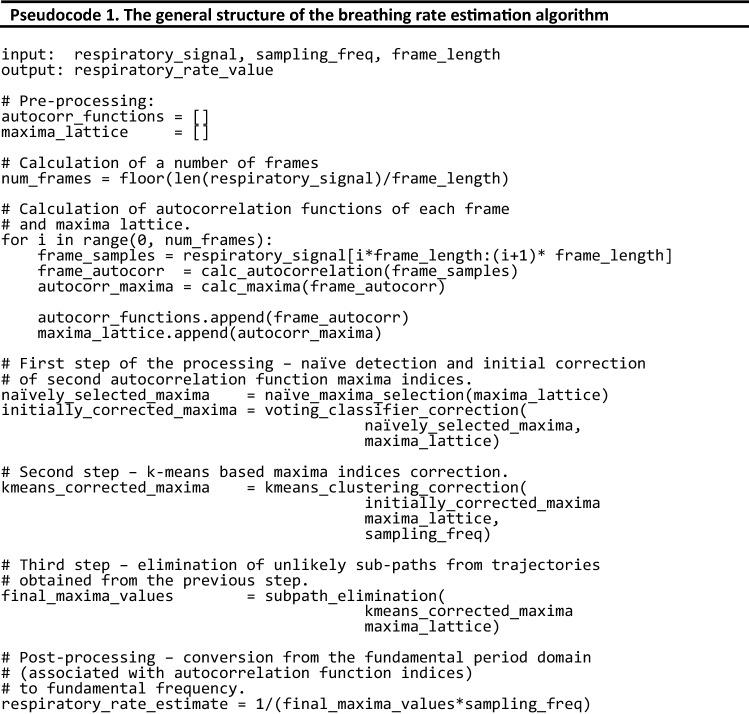


As can be seen in Pseudocode 1, the main activity of the algorithm is comprised of the following three steps:

1. Naïve estimation of the fundamental period of the respiratory signal employing the naïve_maxima_selection function, which calculates the Euclidean distance between the central and all remaining maxima of the autocorrelation function. Next, an initial correction of the respiratory rate estimate is performed using the voting_classifier_correction function, which performs heuristic detection of octave errors using a simple voting classifier.
2. Outlier elimination using kmeans_classifier_correction, which employs the *k*-means clustering algorithm. This leads to the elimination of octave errors that are not detected by the voting classifier used in the previous step. This process is described in more detail later in this section.
3. Elimination of unlikely sub-paths consisting of consecutive autocorrelation function maxima present in the sequence returned by the kmeans_classifier_correction. This is performed by the subpath_elimination function. More details about this process are provided later in this section.

Hence, our algorithm first detects the second-largest maximum in each frame based on the Euclidean distance between the largest maximum (the central one) and every other maximum located in the autocorrelation function. The positions of all the maxima are obtained by analyzing the first and second derivatives of the autocorrelation function. The maximum that is closest to the central one in terms of Euclidean distance (i.e., taking into account the distance on both the *x*-axis and y-axis) is assumed to be the second-largest maximum associated with the fundamental frequency of the signal. Using the Euclidean distance is beneficial because it allows the algorithm to ignore any small maxima positioned between the central peak and second-largest peak, which could be mistakenly marked as the second-largest maximum if only the distance on the *x*-axis is taken into account. Such a situation is shown in Fig. 5.

Additionally, a heuristic voting algorithm has been included to prevent octave errors that, unfortunately, can still occur even when the Euclidean distance-based method is used to detect the second-largest peak. For example, if the Euclidean distance-based method finds that the maximum associated with the fundamental frequency is 1.8-fold larger in 3 or more of the 5 previous detections, it is assumed that the algorithm has made an octave error. In this case, the maximum closest to one in a prior frame will be returned instead of that detected by the algorithm.

The second processing step involves eliminating outliers not detected by the heuristic voting-based algorithm in the previous step. It is based on the *k*-means clustering algorithm, and its structure is defined by Eq. 3. Pseudocode 2 shows its structure and the process.

Let $v=[{v}_{1},{v}_{2}, {\dots , v}_{M}]$ be a set of data in *k*-means_corrected_maxima in ${R}^{N}$ (i.e., each data in a set have *N* features). Then, the *k*-means clustering analysis is performed. The *k*-means clustering problem is to find cluster centers ${C}^{1}$ and ${C}^{2}$ in ${R}^{N}$ such that:

3 $$\underset{{C}_{1}, {C}_{2}}{\mathrm{min}}\sum_{i=1}^{M}\underset{k=\mathrm{1,2}}{\mathrm{min}}\left(\frac{1}{2}{\Vert {v}_{i}-{C}^{k}\Vert }_{2}^{2}\right)$$

The problem is solved equivalently. Given the cluster centers ${C}^{1,t}$ and ${C}^{2,t}$ at iteration *t*, ${C}^{1,t+1}$ and ${C}^{2,t+1}$ at iteration $t+1$ are computed in the following two steps ^52^:

1. ***Cluster assignment***. For each data ${v}_{i}$, assign ${v}_{i}$ to cluster $k(i)$ such that center ${C}^{k\left(i\right),t}$ is nearest to ${v}_{i}$ in the 2-norm.
2. ***Cluster update***. Compute ${C}^{k\left(i\right),t+1}$ as the mean of all points assigned to cluster $k$.

Stop when ${C}^{k,t+1}$=${C}^{k,t}$, $k=\mathrm{1,2}$, else increment $t$ by 1 and go to step 1.
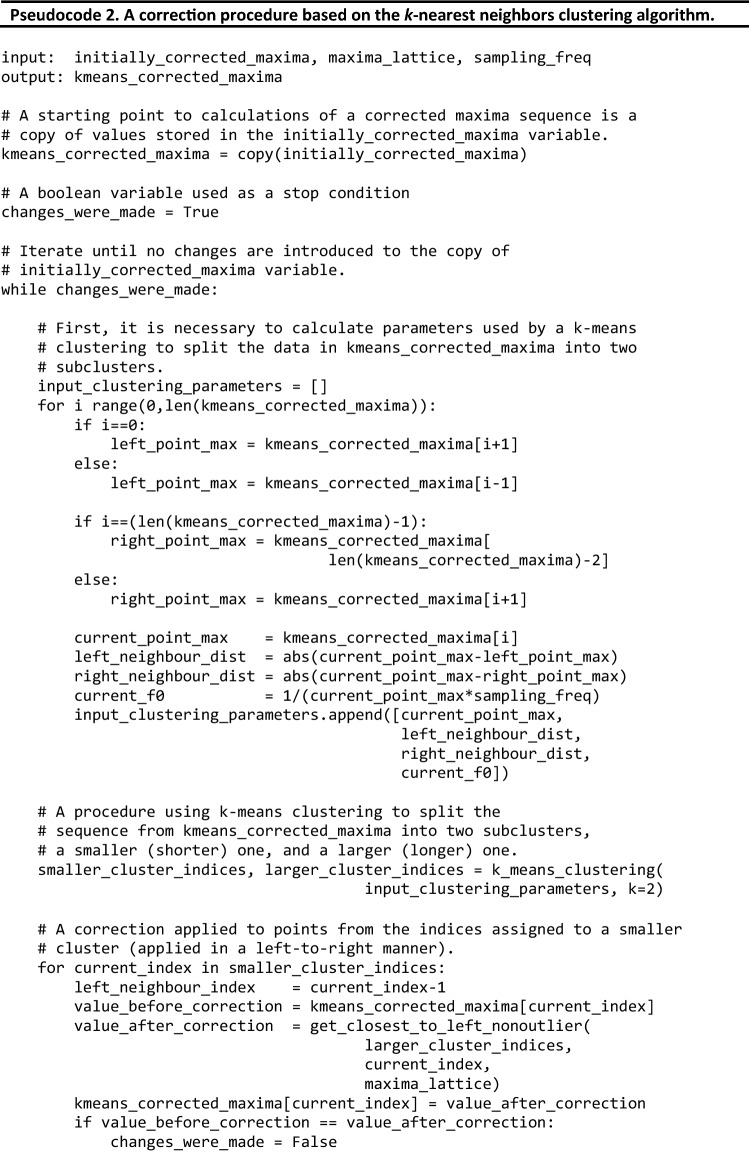


The *k*-means algorithm divides the points on the trajectory into two clusters ($k=2$). The following parameters are employed for clustering:

- current_point_max: The index of maximum on the current trajectory of the autocorrelation function maxima.
- left_neighbour_dist: The distance from the current point on the path to the left neighbor (for the first point on the path, it is assumed to be equal to the distance to the right neighbor).
- right_neighbour_dist: The distance from the current point on the path to the right neighbor (for the last point on the path, it is assumed to be equal to the distance to the left neighbor).
- current_f$\emptyset$: The fundamental frequency value associated with the current index of maximum on the tracked autocorrelation maxima path.

**Figure 8:**
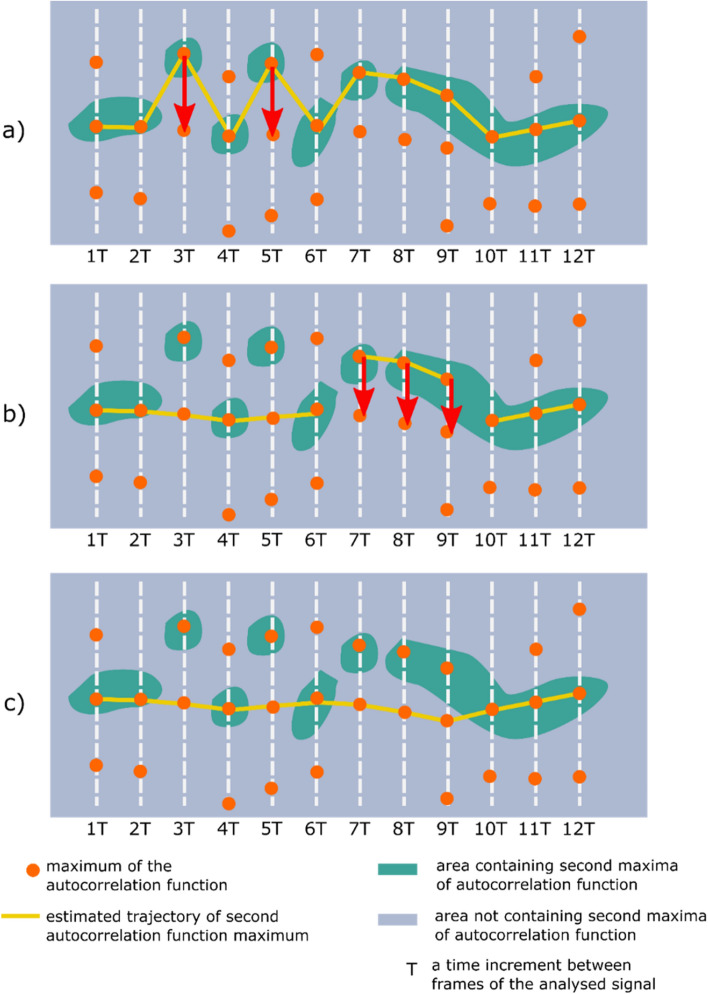
Graphical illustration of two refinement operations applied to a second autocorrelation function trajectory obtained in a naïve manner with the Euclidean-based approach. (**a**) Outlier elimination performed with the *k*-means algorithm. (**b**) Processing based on detecting transitions between points on a lattice that are not the nearest neighbors of each other. (**c**) The final result obtained with the proposed heuristic f0 (fundamental frequency) tracking algorithm.

From the two clusters returned by the *k*-means clustering, the smaller cluster is assumed to be the cluster of outliers. Next, each outlier point on the trajectory is changed in the left-to-right direction (on the x-axis) to move to the maximum closest (on a lattice of maxima such as in Figs. 6 and 7) to the non-outlier maximum from the preceding non-outlier frame. A get_closest_to_left_nonoutlier procedure is used for this operation (see Pseudocode 2). Its principle of operation is shown in more detail in Fig. 8a, where the red arrows show the movements of the outlier points based on the changes made by the *k*-means algorithm. The *k*-means algorithm from the Scikit-learn (version 0.24.1) Python library is used. This process is repeated until all points classified in the “outliers” cluster are closest neighbors to the maxima found in the preceding frames. The second stage of the heuristic respiratory rate estimation algorithm is then complete, and there are no further changes to the positions of the points on the tracked paths.

The third and final processing step involves dividing the tracked path of the autocorrelation function maxima into subsegments. The original path is split into points where the transition is made, but not between the two closest maxima of the two neighboring frames, as such a situation may still occur even after the previous step. An example of such a case is shown in Fig. 8b,c shows the final result of the proposed heuristic f0 tracking algorithm.

**Figure 9:**
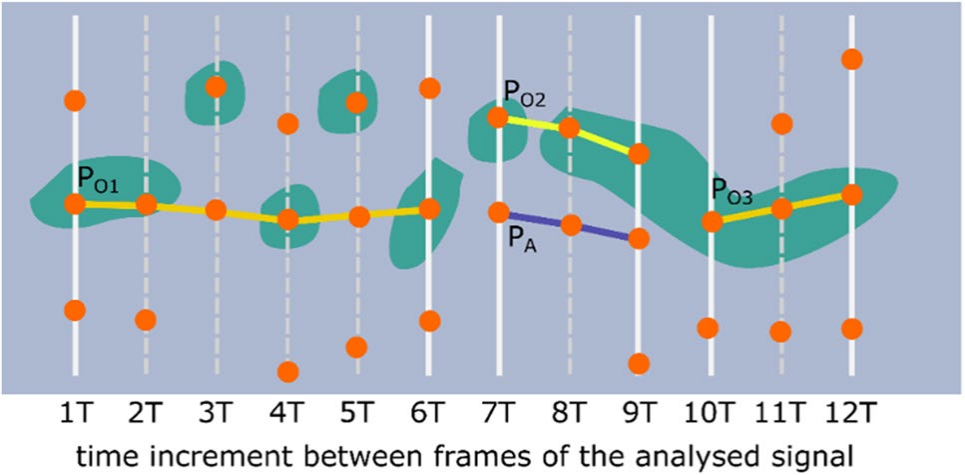
Graphical illustration of the path depicted in Fig. 8 after it has been split into sub-paths (${P}_{O1}$, ${P}_{O2}$, and ${P}_{O3}$). Also, a possible alternative path for ${P}_{O2}$ is shown (depicted as ${P}_{A}$).

After the path is split into subsegments, they are merged in a left-to-right order. This process involves analyzing pairs of original subsegments and possible alternatives to one of the segments present in each pair. A detailed description of the path merging process is presented next, based on the example shown in Fig. 9.

**Figure 10:**
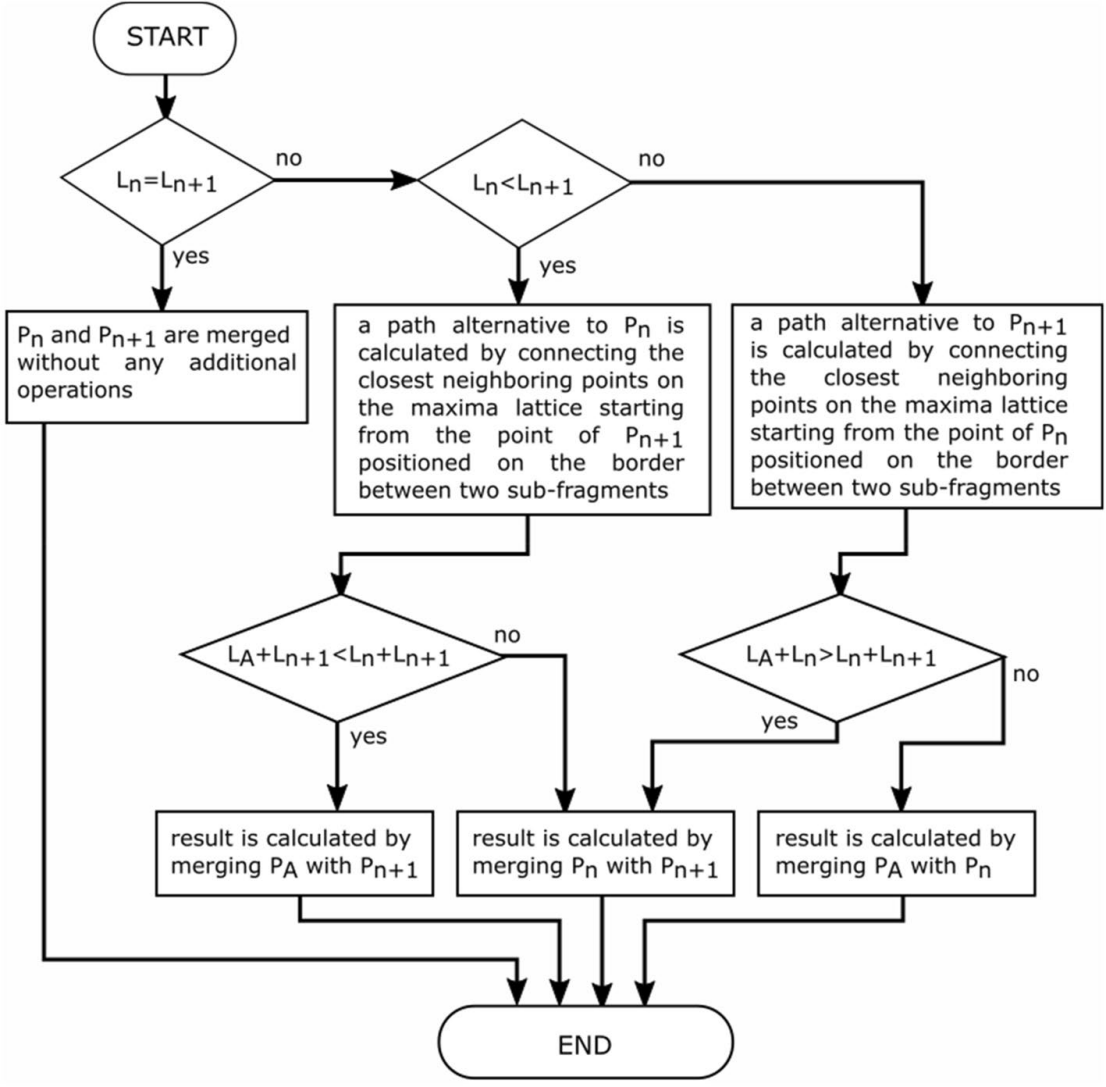
The decision diagram for a single iteration in the path merging process. ${P}_{n}$ and ${P}_{n+1}$ denote two currently considered sub-paths (e.g., ${P}_{O1}$ and ${P}_{O2}$ in the first iteration of the case shown in Fig. 9).

In Fig. 9, the original path segments are denoted as ${P}_{O1}$, ${P}_{O2}$, and ${P}_{O3}$. An alternative to path ${P}_{O2}$ is ${P}_{A}$. ${L}_{O1}$, ${L}_{O2}$, ${L}_{O3}$, and ${L}_{A}$ denote the lengths of the paths. The merging process is performed in an iterative manner starting from the leftmost pair of path sub-fragments. The two sub-paths considered in each iteration are denoted as ${P}_{n}$ and ${P}_{n+1}$ with lengths ${L}_{n}$ and ${L}_{n+1}$, respectively. A single merger iteration is calculated according to the algorithm depicted in Fig. 10.

For the example shown in Fig. 9, first, the pair of ${P}_{O1}$ and ${P}_{O2}$ is considered, and the result is a merged path denoted as ${P}_{M1}$. Next, the pair of ${P}_{M1}$ and ${P}_{O3}$ is considered, and the result of this second iteration is the final result of the path sub-fragments merging algorithm.

**Figure 11:**
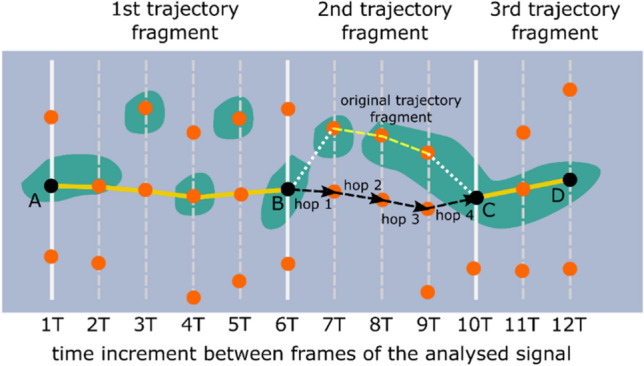
Example of the operations performed during the third stage of the respiratory rate estimation.

An example of this process is depicted in Fig. 11, which shows a sample trajectory split into three fragments. The first merger is performed between the AB fragment (the larger one) and the BC fragment (the shorter one, marked with a white dashed line). The alternative path is obtained by hopping between the closest points on a maxima lattice starting from point B, which is a common point for the AB and BC paths. In this case, the trajectory calculated in 5 hops between closest neighbors turns out to be shorter than the original trajectory; therefore, the algorithm will choose this alternative path instead of the original one for a merger. The next merger will be performed between the newly calculated AC fragment and the CD fragment. In this case, the CD trajectory is the shortest one to reach point D; therefore, no changes are needed. This process repeats until there are no more sub-paths to merge. The final result of the third stage of processing is also the final result of the whole heuristic algorithm for estimating the respiratory rate.

**Figure 12:**
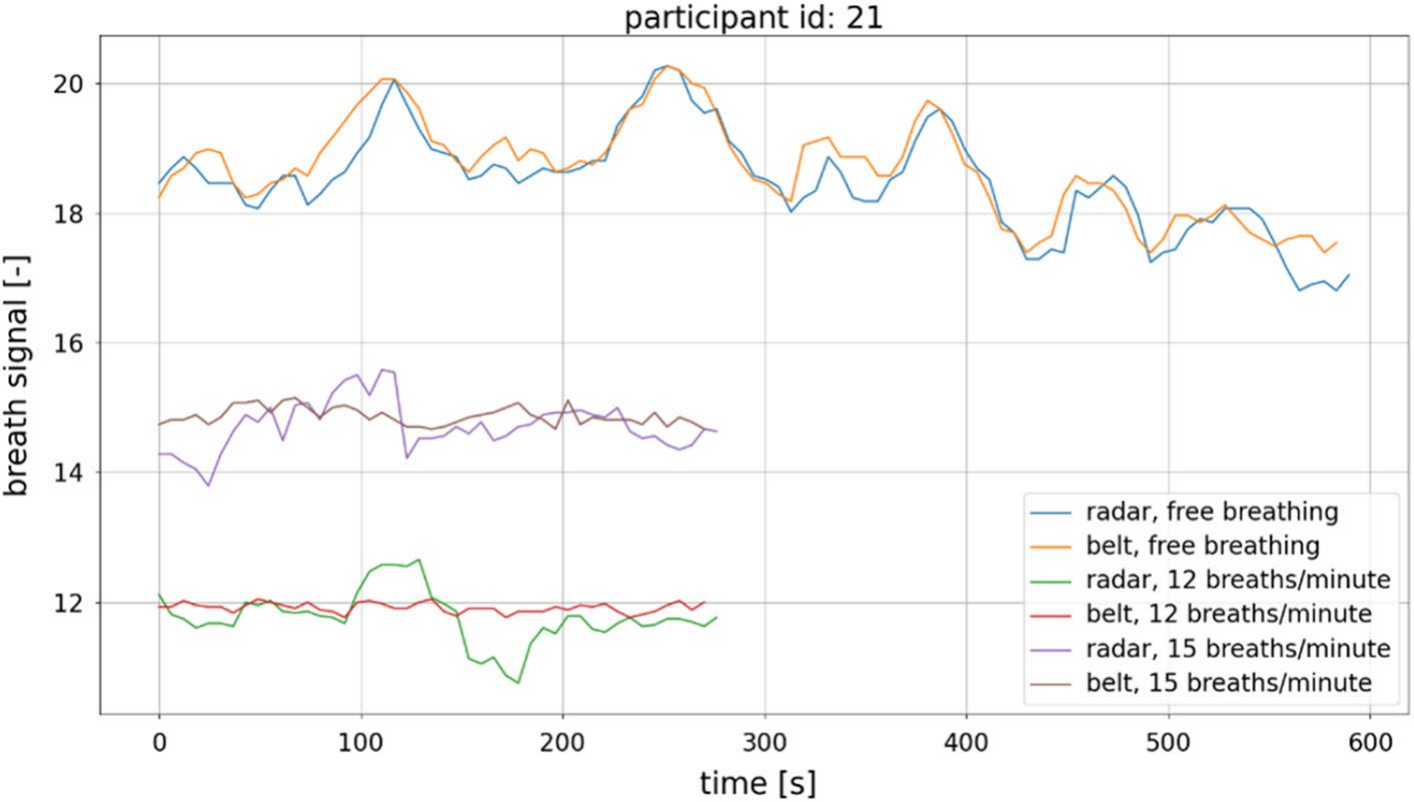
Examples of breathing rate estimations obtained for Participant 21.

At this point, the only post-processing operation to be performed is the calculation of the fundamental period and consequently the calculation of the fundamental frequency from the consecutive indices of the autocorrelation function maxima. Examples of respiratory rate estimations obtained with the algorithm from the experimental data are shown in Fig. 12.

### Ethical approval

All procedures performed in studies involving human participants were in accordance with the ethical standards of the institutional and/or national research committee and with the 1964 Helsinki Declaration and its later amendments or comparable ethical standards.

### Informed consent

Informed consent was obtained from all individual participants included in the study.

## Results and discussion

**Figure 13:**
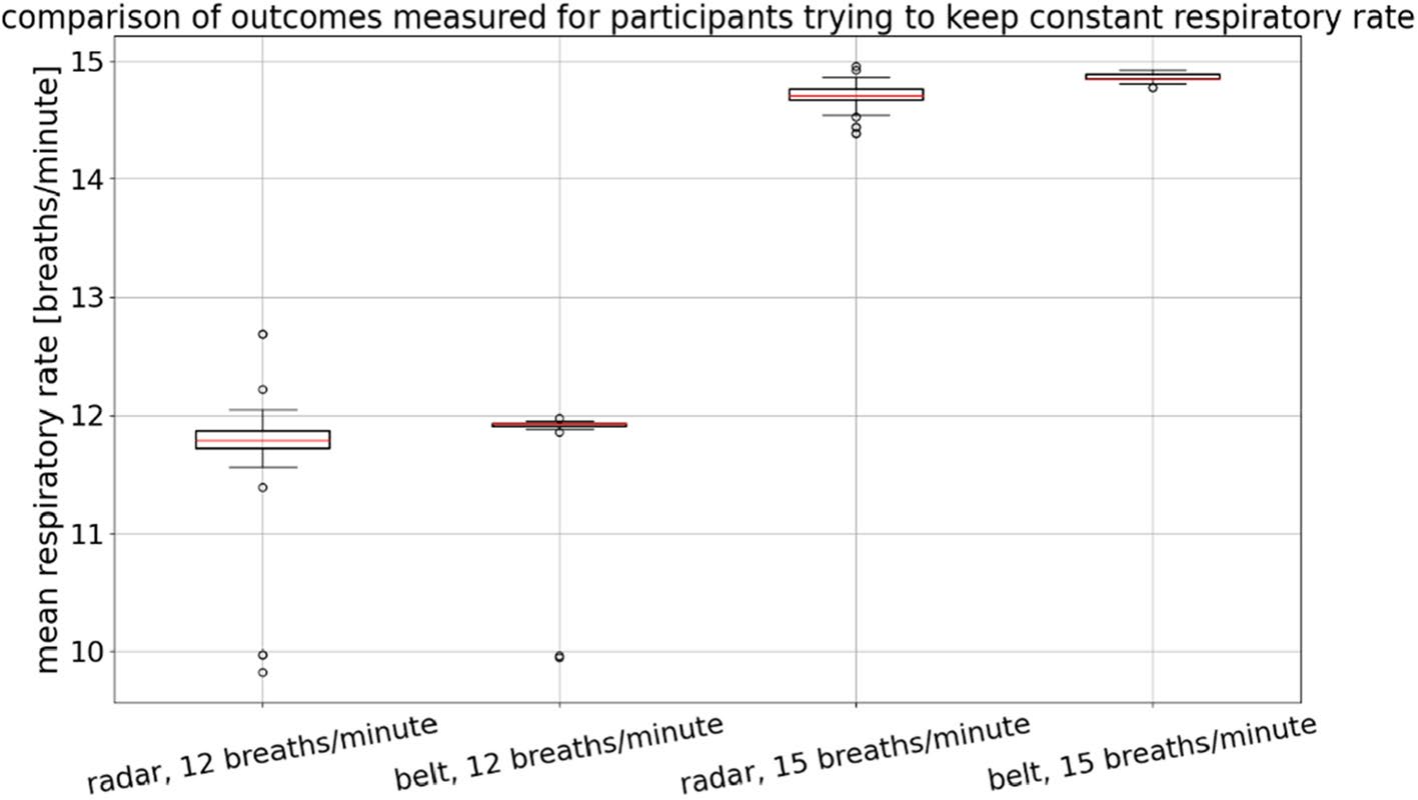
Estimated respiratory rates of participants who were asked to maintain a constant breathing rate. For each group, n = 31, as each participant contributed once to each group depicted in the figure.

Each participant’s breathing rate was estimated for each of the experimental scenarios outlined in the protocol. Figure 13 shows the estimated respiratory rates obtained with the belt and radar-based sensors when the participants were asked to maintain a constant respiratory rate of either 12 or 15 breaths/min.

These data were subjected to statistical analysis. The commonly used value of 0.05 was used as the significance level $\alpha$. Levene’s test was performed to determine whether the variances among the groups shown in Fig. 13 are equal, and the resultant test statistic was 3.60, corresponding to a *p*-value of 0.02. Hence, it was concluded that the variances among the groups shown in Fig. 13 are unequal. Therefore, the Kruskal–Wallis test was used to test the equality of medians between pairs of groups shown in Fig. 13. The Kruskal–Wallis test statistic was found to be 103.32, with a *p*-value less than $.001,$ leading to the conclusion that at least one pair of groups has a statistically significant difference between their medians. Finally, Dunn’s post hoc test was conducted to determine which groups were different, and the results are shown in Table 1.

**Table 1:**
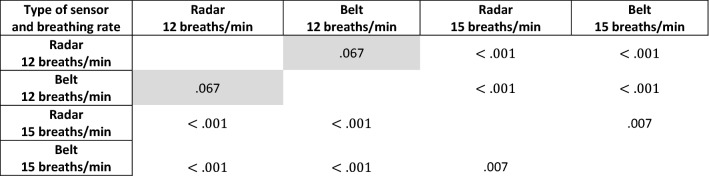
Dunn’s post hoc test results for the scenarios in which participants were asked to maintain a constant breathing rate. Statistically insignificant values are marked with a gray background.

The median breathing rates determined under each condition were as follows: 11.79 breaths/min (radar, 12 breaths/min), 11.92 breaths/min (belt, 12 breaths/min), 14.71 breaths/min (radar, 15 breaths/min), and 14.85 breaths/min (belt, 15 breaths/min). The difference between the radar- and belt-derived estimates at 12 breaths/min was found to be statistically insignificant. There was also a statistically significant difference between the estimates at 15 breaths/min; however, the difference was only 0.14 breaths/min. In terms of practical implementation, such a difference may be negligible.

Additionally, we performed statistical analysis on the output of the first step of the proposed algorithm—the fundamental frequency estimation generated using the Euclidean distance method and the algorithm from a NeuroToolkit2 Python library^53,54^. In the case of the Euclidean distance-based algorithm, no heuristics were used (including the voting classifier). The NeuroToolkit2 library algorithm employs simple peak detection performed in the time domain, and *rsp_clean* and *rsp_peaks* functions are used for calculation. Detailed descriptions of the time-domain peak detection algorithm can be found in^53,54^. The median respiratory rate, interquartile range (IQR), and standard deviation (SD) are shown in Table 2, along with the corresponding statistical measures resulting from the use of the naïve and time-domain peak-finding algorithms only.

**Table 2:** Respiratory rate estimates calculated using the proposed algorithm, the naïve version of the proposed algorithm (using the Euclidean distance to find the second peak of the autocorrelation function), and a time-domain peak-finding algorithm from the NeuroKit2 Python library^54^. The measure of central tendency is the median value, and the variance measures are the interquartile range (IQR) and standard deviation (SD).

	Proposed algorithm			Naïve (Euclidean) algorithm			Time-domain peak-finding algorithm		
	Median	IQR	SD	Median	IQR	SD	Median	IQR	SD
Radar 12 breaths/min	11.79	0.14	0.63	11.79	0.25	0.6	17.58	5.86	3.44
Belt 12 breaths/min	11.92	0.02	0.48	11.92	0.02	0.48	8.79	0.37	0.76
Radar 15 breaths/min	14.71	0.09	0.14	14.71	0.14	0.37	14.65	7.32	3.77
Belt 15 breaths/min	14.85	0.04	0.04	14.85	0.06	0.04	11.72	0	0.43

The proposed algorithm and its simplified version achieved identical performance in terms of median respiratory rate. However, the additional processing performed by the more complex proposed algorithm resulted in a reduction in the IQR and standard deviation (in most cases). Therefore, one can conclude that both versions of the algorithm can be used in practice. The more complex proposed version could be used in situations where reduced variance in estimate values is needed. The simple time-domain algorithm was found to be notably less precise than the algorithms employing the autocorrelation function.

Participants were also monitored when they were breathing freely, that is, without maintaining a particular respiratory rate. While analysis of such data is possible, it is more complex than analyzing data obtained in a constant respiratory rate scenario. In this case, a similarity measure between respiratory rate estimates that is a function of the acquisition time is required, and this must be calculated before results can be compared (see Fig. 12). An example of such a measure is distance, which is calculated using a dynamic time warping (DTW) algorithm and is often used as a similarity measure for time series^55^.

**Figure 14:**
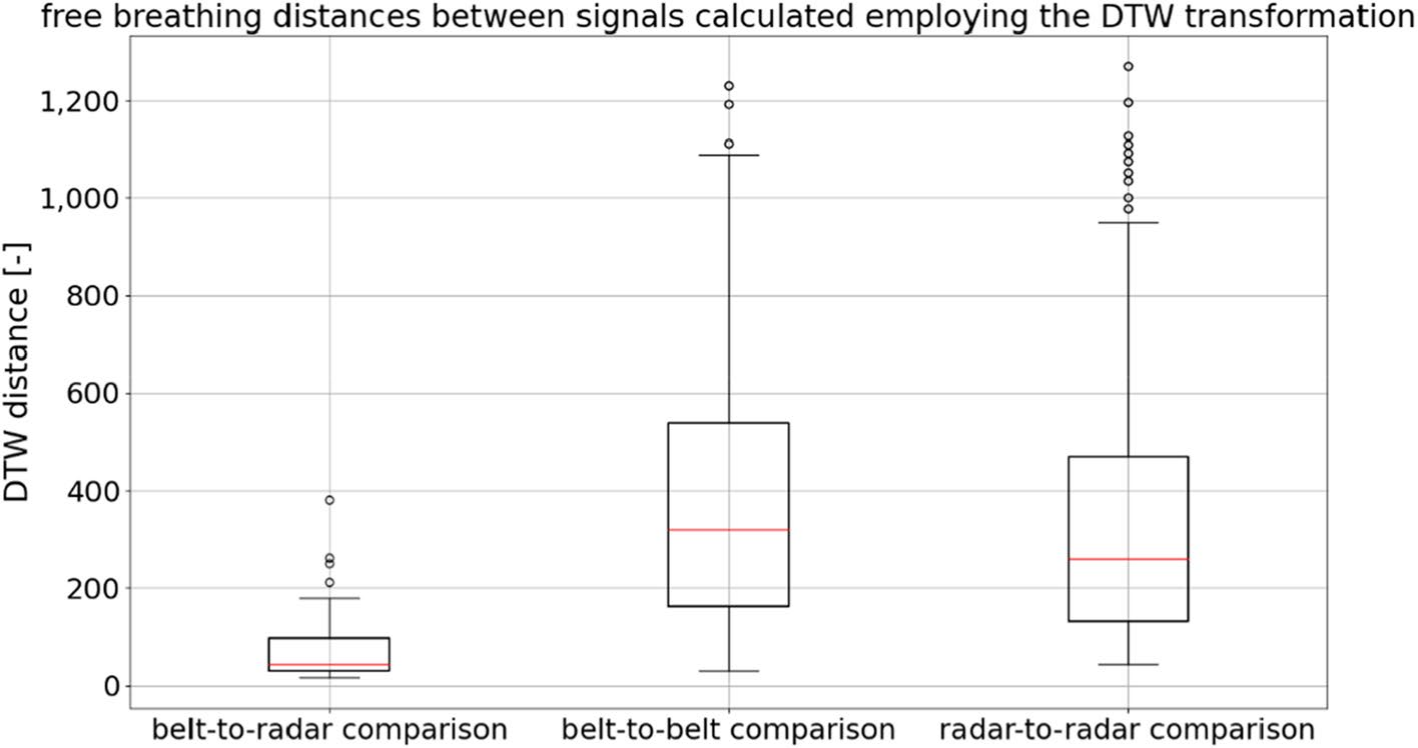
A dynamic time warping (DTW)-based algorithm was used to calculate the DTW distance in three different situations. Belt- and radar-derived data from the same participant were compared, as well as belt-derived data from different participants and radar-derived data from different participants.

For this purpose, we implemented an algorithm provided in the FastDTW Python library (version 0.3.4). Distances were calculated for three cases. In the first case, data from one person were analyzed, and 31 comparisons were made between measurements obtained from the belt and from the microwave-based radar sensor. In the second case, 930 comparisons were made between measurements obtained from pairs of different people using the belt. In the third case, 930 comparisons were made between measurements obtained from pairs of different people using the microwave-based radar sensor. The results are depicted in the form of a boxplot in Fig. 14.

In Fig. 14, it can be clearly seen that the variation in the measurements is less when different devices are used for one participant than when the same device is used for different participants. Statistical analysis was conducted to further investigate the observed differences. The Python libraries used in the analysis of the constant breathing rate data were also used here. The significance level was assumed to be 0.05. Levene’s test returned a result of 13.43, which resulted in a *p*-value smaller than 0.001. Therefore, the variances among the groups shown in Fig. 14 were found to be not equal, and the Kruskal–Wallis test was performed to compare the medians of the groups in Fig. 14. The Kruskal–Wallis test returned a result of 74.21; thus, the *p*-value of this test was also less than 0.001. Therefore, it was concluded that there are significant differences between the medians of the groups depicted in Fig. 14. Finally, Dunn’s post hoc test was performed, and the resulting matrix of *p*-values is shown in Table 3.

**Table 3:** Dunn’s post hoc test results for DTW-based distances between respiratory rate estimates measured using the belt and radar-based sensors (all differences were found to be statistically significant).

Comparison type	Belt-to-radar	Belt-to-belt	Radar-to-radar
Belt-to-radar		$<.001$	$<.001$
Belt-to-belt	$<.001$		.008
Radar-to-radar	$<.001$	.008	

The observed median distance for the belt-to-radar comparison was found to be 43.99, the lowest of the observed values. The median distance for the belt-to-belt comparison was found to be 320.14, and the median distance for the radar-to-radar comparison was found to be 260.94.

## Conclusions

We have developed, tested, and described an improved method for measuring the respiratory rate using a microwave radar-based sensor device. Importantly, this device offers a contactless way to estimate the breathing rate, which is vital when managing patients with highly contagious, infectious diseases. It is also crucial to accurately assess the respiratory rate. Hence, we have developed a heuristic algorithm that provides a precise estimate even when misleading autocorrelation function maxima are present, which can potentially cause erroneous detections if a simpler algorithm is used.

The findings show that the proposed algorithm and microwave radar-based sensor performed at a similar level to a standard wearable belt in terms of respiratory rate monitoring. It was also observed that our contactless radar-based device is a viable alternative to the belt-based device for analyzing both breathing at a constant rate and free breathing. The breathing rate estimates obtained using data from the two different types of sensors were found to be similar in a statistical sense, as shown by the findings of the constantly controlled breathing rate scenario. The main source of variability in the measurement outcomes was found to be the person-to-person differences in breathing. Switching from radar- to belt-based measurement and vice versa resulted in significantly less difference in the measured respiratory rate estimate, as shown in the analyses of the signals captured when participants were instructed to breathe freely.

Through the experimental and statistical analyses presented here, we have shown that our Respiration and Circulation Monitor has the potential as a viable and accurate respiratory rate monitoring device. It can be used as a replacement for the belt device, which requires the expertise of an experienced medical consultant for its use. In contrast, our contactless device is a compelling alternative for the estimation of respiratory rate, especially when direct contact with the subject should be limited.

The monitor has potential applications in cardiopulmonary monitoring. In situations of mass illness, such as the COVID-19 pandemic, it can be used for: monitoring quarantined and asymptomatic patients residing at home; diagnosis in specialist clinics, especially those with an internal medicine focus; diagnosis in hospital emergency departments for cases with indications for hospital admission; hospitalized patients where minimal contact with hospital staff and equipment is required to detect clinical deterioration, indications for mechanical ventilation, or assessment of the possibility of discharge from the intensive care unit are required. In non-pandemic situations, it can be used for diagnosing and monitoring patients with heart failure after stroke (including at home); improved assessment of abnormal breathing during sleep (including at home); research on respiratory rhythms and patterns and respiratory and cardiovascular regulation, and with large cohorts; and improved monitoring of patients participating in clinical trials.
